# Increased type-IV collagenase (MMP-2 and MMP-9) activity following preoperative radiotherapy in rectal cancer

**DOI:** 10.1054/bjoc.1999.1025

**Published:** 2000-02

**Authors:** A Kumar, H M Collins, J H Scholefield, S A Watson

**Affiliations:** Academic Unit of Cancer Studies, Division of GI Surgery, University Hospital, Nottingham, NG7 2UH, UK

**Keywords:** rectal cancer, radiotherapy, matrix metalloproteinase, type-IV collagenases

## Abstract

The aim of this study was to investigate the effect of preoperative high-dose radiotherapy (25 Gy in 5 fractions over 5 days) on the type-IV collagenase protein profile, in patients with resectable rectal cancer, by gelatin zymography. Biopsy samples of tumour and distant normal mucosa from 12 patients with resectable rectal cancer were obtained pre- and post-radiotherapy. Expression of type-IV collagenases (both pro- and active forms) was studied using gelatin zymography. Enzyme levels were normalized for total protein content of each sample. Rectal cancer specimens expressed both pro (72 kDa) and active (62 kDa) forms of MMP-2 but only the pro form of MMP-9 (92 kDa). Normal mucosa showed expression of the pro forms of MMP-2 and MMP-9 while no active form of either enzyme was detected in any of the samples. A significant three- to fourfold increase (*P*< 0.01) of active matrix metalloproteinases (MMP)-2 (62 kDa) was seen in malignant rectal mucosa after radiotherapy. The effect of radiotherapy also led to a twofold increase (*P* = 0.047) of pro MMP-2 (72 kDa) and a two- to threefold increase (*P* = 0.03) of the precursor form of MMP-9 (92 kDa). In contrast, in normal mucosa expression of the precursor form of MMP-9 (92 kDa) did not change after radiation, and no significant effect on the levels of pro MMP-2 (72 kDa) was observed. Preoperative high-dose radiotherapy leads to an increase in activity of type-IV collagenases in patients with resectable rectal cancer. Type-IV collagenase inhibition may be a useful therapeutic adjunct to radiotherapy in rectal cancer. © 2000 Cancer Research Campaign


*British Journal of Cancer* (2000) **82**(4), 960–965
© 2000 Cancer Research Campaign
DOI: 10.1054/ bjoc.1999.1025, available online at http://www.idealibrary.com on IDEAL®

### Increased type-IV collagenase (MMP-2 and MMP-9) activity following preoperative radiotherapy in rectal cancer

**A Kumar, HM Collins, JH Scholefield and SA Watson**

Academic Unit of Cancer Studies, Division of GI Surgery, University Hospital, Nottingham NG7 2UH, UK

**Summary** The aim of this study was to investigate the effect of preoperative high-dose radiotherapy (25 Gy in 5 fractions over 5 days) on the type-IV collagenase protein profile, in patients with resectable rectal cancer, by gelatin zymography. Biopsy samples of tumour and distant normal mucosa from 12 patients with resectable rectal cancer were obtained pre- and post-radiotherapy. Expression of type-IV collagenases (both pro- and active forms) was studied using gelatin zymography. Enzyme levels were normalized for total protein content of each sample. Rectal cancer specimens expressed both pro (72 kDa) and active (62 kDa) forms of MMP-2 but only the pro form of MMP-9 (92 kDa). Normal mucosa showed expression of the pro forms of MMP-2 and MMP-9 while no active form of either enzyme was detected in any of the samples. A significant three- to fourfold increase ($P < 0.01$) of active matrix metalloproteinases (MMP)-2 (62 kDa) was seen in malignant rectal mucosa after radiotherapy. The effect of radiotherapy also led to a twofold increase ($P = 0.047$) of pro MMP-2 (72 kDa) and a two- to threefold increase ($P = 0.03$) of the precursor form of MMP-9 (92 kDa). In contrast, in normal mucosa expression of the precursor form of MMP-9 (92 kDa) did not change after radiation, and no significant effect on the levels of pro MMP-2 (72 kDa) was observed. Preoperative high-dose radiotherapy leads to an increase in activity of type-IV collagenases in patients with resectable rectal cancer. Type-IV collagenase inhibition may be a useful therapeutic adjunct to radiotherapy in rectal cancer. © 2000 Cancer Research Campaign

**Keywords:** rectal cancer; radiotherapy; matrix metalloproteinase; type-IV collagenases

For rectal carcinoma, the incidence of local recurrence following curative resection ranges between 4 and 32% with a median of over 15% (Abulati and Williams, 1994). The relatively high incidence of recurrence and poor overall prognosis following apparently curative surgery for rectal carcinoma has led to the increasing use of adjuvant preoperative radiotherapy in Europe. Most of the studies (Manchester Trial 1994; Stockholm 1 Trial, 1995; Swedish Rectal Cancer Trial, 1997) using a higher dose of radiation equivalent to 25 Gy or more, with variable fraction schedules, have demonstrated a significant reduction in local recurrence compared with surgery alone. Matrix metalloproteinases (MMPs) are a family of zinc-containing enzymes (Murphy et al, 1992; Stetler-Stevenson et al, 1993) involved in the degradation of different components of the extracellular matrix. There is considerable evidence to indicate that individual MMPs have important roles in both tumour invasion and spread (Davies et al, 1993*a*; Muller et al, 1993; Urbanski et al, 1993; Boag et al, 1994). MMPs have been classified into collagenases, gelatinases, stromelysins and the membrane-type MMPs. In particular, the gelatinases or type-IV collagenases (MMP-2 and MMP-9) have been implicated in playing a significant proteolytic role in colorectal cancer invasion and metastasis (Poulson et al, 1992; Pyke et al, 1993; Zucker et al, 1993; Jaziorka et al, 1994). A moderate increase in the expression of pro MMP-2 during the adenoma-carcinoma sequence has been reported and contrasts with the level of active MMP-2, which is absent in normal tissue but increases dramatically on conversion to the malignant phenotype (Parsons et al, 1998). This evidence supports the view of other researchers (Yamagata et al, 1991; Liabakk et al, 1996) that activation of MMP-2 is a crucial step in tumour invasiveness. It has been described that poor prognosis in a range of human cancers correlated with increased activity of active MMP-2 and pro MMP-9 (Azzam et al, 1993; Seir et al, 1996). Zeng et al (1996) demonstrated that levels of MMP-9 were associated with a significant shorter disease-free and overall survival in patients with colorectal carcinoma. They also demonstrated that high MMP-9 mRNA expression progressively increased from early colorectal carcinoma through invasive and metastatic stages. Their results supported the observations based on in vitro (Turpeenniemi-Hujanen et al, 1985; Ballin et al, 1988; Yamagata et al, 1988; Moll et al, 1990) and animal data (Nakajima et al, 1990) that high tumour MMP-9 expression is associated with increased metastatic potential. There is considerable evidence suggesting that these enzymes also play a role in the growth of primary and secondary tumours (reviewed by Chambers and Matrisian, 1997; Duffy and McCarthy, 1998), including the process of angiogenesis (Johnson et al, 1994).

In vitro studies have shown that irradiation leads to induction of MMPs in different types of cell lines (Sheela et al, 1986; Sawaya et al, 1994). Using gelatin zymography, Sawaya et al (1994) demonstrated that after single-dose (12 Gy) irradiation of rat astrocytes, there was a continuous increase in the levels of pro MMP-2 (72 kDa) at different time intervals, with a significant fivefold increase at 48 h after irradiation. They proposed that high levels of MMP-2 along with changes in tissue plasminogen activator (tPA) may be responsible for radiation-induced damage to the central nervous system following radiotherapy of brain tumours. These

*Received 5 July 1999*
*Revised 13 September 1999*
*Accepted 22 September 1999*

*Correspondence to:* A Kumar observations led us to hypothesize that preoperative high-dose radiotherapy leads to overexpression of type-IV collagenases in rectal cancer. This may be responsible for the promotion of angiogenesis and in the re-establishment of invasion of remaining viable cancer cells in an attempt to resurrect the growth potential of the tumour. The aim of this study was to determine the effect of preoperative high-dose short-term radiotherapy (25 Gy in 5 fractions in 5 days) on the type-IV collagenase protein profile of rectal cancer.

### PATIENTS AND METHODS

Twelve patients with resectable adenocarcinoma of the rectum, located within 15 cm from the anal verge, were included in the study. All patients underwent standard preoperative radiotherapy, which was delivered to the pelvis with a three-beam technique. The dose delivered was 25 Gy in 5 fractions over 5 days. Surgery in the form of anterior resection or abdomino-perineal resection was performed within a week following completion of the radiotherapy. This study was carried out with ethical committee approval and all patients provided written informed consent to participate in the study.

### Tissue samples

Biopsy samples of tumour ($n$ = 24) and distant normal mucosa ($n$ = 14) were obtained pre- and post-radiotherapy. Specimens were obtained from the tumour edge at different sites under vision, avoiding the necrotic centre. Multiple specimens from the same tumour were pooled to give a uniform representation of the tumour. The post-radiotherapy biopsies were performed 48 h after completion of radiotherapy and just before surgery. The post-radiotherapy biopsies were taken from sites close to the preradiotherapy biopsy sites and the normal mucosa was obtained from within the radiation field. The reason for performing the post-irradiated biopsy rather than obtaining surgical specimens, was to eliminate the changes in MMP expression induced by surgical stress and ischaemia. There were no complications associated with either biopsy procedure in any of the patients. All tissue samples were cryopreserved in liquid nitrogen, following their removal from the patients, and stored at –80°C until use. Ten and 30 micron cryostat sections were cut from each tissue sample for histology and electrophoretic analysis respectively. Haematoxylin and eosin staining was performed on each section to validate the presence of tumour epithelium and stroma in tumour samples and normal epithelium. For zymography between 10 and 20 mg of tissue was used for each sample, as earlier work had shown this quantity of tissue to give visible bands within the linear range of the zymogram. Sample buffer (sodium dodecyl sulphate (SDS) 100 μl) was added to each tissue sample in an Eppendorf and homogenized. The sample was then centrifuged at 16 000 rpm for 5 min and 25 μl of the supernatant was micropipetted into the wells of a precast zymogram gel. The remaining sample was used to determine the protein content. Type IV collagenase levels were expressed per microgram (μg) of protein.

### Gelatin zymography

Gelatin zymography was performed as previously described (Heussen and Dowdle, 1980; Brown et al, 1993) using commercially available 10% acrylamide precast zymogram gels containing 0.1% gelatin (Novex, GMBA Germany). Pure type-IV collagenase samples were obtained from British Biotech and prepared from transfected Chinese hamster ovary cells (Chandler et al, 1995). Western blotting using monoclonal antibodies for pro and active MMP-2 and MMP-9 was performed previously to verify that the bands seen on zymography were as described (data not shown). All tissue samples were run in duplicate with pre- and post-irradiated samples studied on the same gel to eliminate inter-gel variation as a source of error when comparing expression of type-IV collagenases in pre and post radiotherapy tissues. The two outer lanes contained pure type-IV collagenase (72 kDa and 92 kDa) standards at 5 ng concentration. After electrophoresis, the gels were rinsed in 2.5% Triton X-100 for 30 min and incubated at 37°C for 18 h in developing buffer (50 mM Tris–HCl buffer, pH 7.5, containing 5 mM calcium chloride, 0.2 M sodium chloride). The gels were stained with 0.05% colloidal Coomassie blue and destained in 30% methanol and 10% acetic acid in water. Gelatinolytic enzymes were detected as transparent bands on the blue background of the Coomassie-blue-stained slab gel. Quantification was performed using a flat bed scanner and the Apple Macintosh software, Adobe Photoshop and NIH Image Analysis Package as previously described (Davies et al, 1993*b*; Kleiner and Stetler-Stevenson, 1994). The bands were scanned in two positions by densitometry and the peak areas were averaged to give the enzymatic activity.

### Protein determination

The residual 50 μl of each sample was centrifuged at 4°C at 16 000 rpm for 5 min. The protein was determined in the samples using a Pierce Kit (Rockford, IL, USA). The standard was bovine serum albumin (BSA) which was made up in Tris-buffered saline (TBS, pH 7.6) giving a concentration range of 0.2–1.2 mg per ml. Buffer, 10 μl of BSA standard or sample was then pipetted into each well. Protein assay reagent was added (200 μl per well). The plate was covered and incubated at 37°C for 30 min and the absorbance read at 562 nm on the plate reader.

### Immunocytochemistry

The cellular localization of enzymes was performed using anti-MMP-2 and anti-MMP-9 (R&D) on all tissue sections.

### Statistical analysis

All data was found to be non-parametrically distributed and therefore $P$-values for comparison of enzyme levels were calculated using the Wilcoxon paired test and the Mann–Whitney $U$-analysis. Median values of the enzyme levels were used in these analyses because of limited sample numbers.

### RESULTS

Twelve fresh frozen rectal cancers and seven samples of rectal mucosa from the same patients, pre- and post-radiotherapy were evaluated by gelatin zymography. Haematoxylin and eosin staining confirmed both malignant epithelium and stroma in all the cancer specimens and normal epithelium and stroma in all the normal mucosal specimens. Computer-generated image analysis of malignant or normal epithelium/stroma was performed and the

© 2000 Cancer Research Campaign

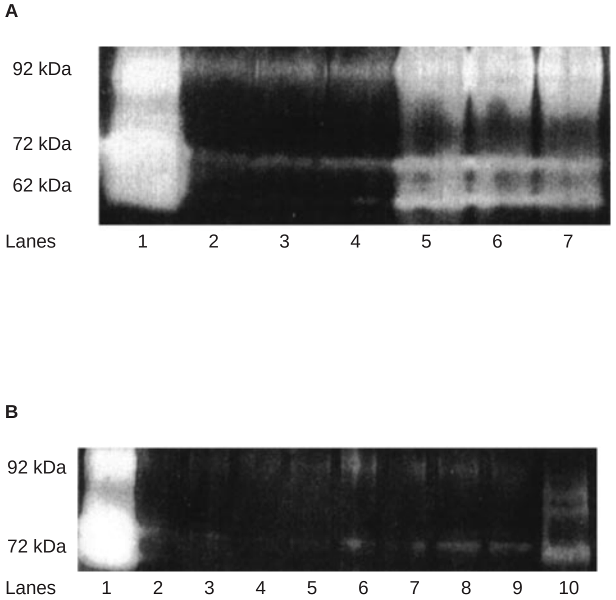

**Figure 1** (**A**) Gelatin zymography of rectal cancer specimens (patient no. 10) showing expression of type-IV collagenases pre- (lanes 2–4) and post-radiotherapy (lanes 5–7). Lane 1 shows molecular weight standards. (**B**) Gelatin zymography showing expression of type-IV collagenases in pre- (lanes 2, 3, 6, 7) and post-radiotherapy (anes 4, 5, 8, 9) normal mucosal specimens. Lanes 1 and 10 show molecular weight standards

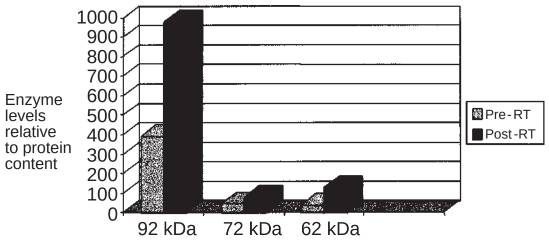

**Figure 2** Bar chart comparing median values of 92 kDa ($P = 0.03$), 72 kDa ($P = 0.047$) and 62 kDa ($P < 0.01$) enzymes (measured as square pixels per microgram of protein) in pre- and post-radiotherapy rectal cancer specimens ratio was found to be comparable in pre- and post-radiotherapy sections.

### Rectal cancer

All rectal cancers expressed pro MMP-9 (92 kDa) before radiotherapy. Pro MMP-2 (72 kDa) was detected in 9/12 samples, active MMP-2 (62 kDa) in 8/12 samples with virtually no expression of the active MMP-9 (82 kDa) enzyme, preradiotherapy (Table 1). Figure 1A shows expression of type-IV collagenases in a selected rectal cancer specimen pre- and post-radiotherapy.

Following radiotherapy, there was a two- to threefold increase in the levels of pro MMP-9 and this difference was statistically significant ($P = 0.03$, Figure 2). No sample showed detectable levels of active MMP-9, either pre- or post-radiotherapy. Pro MMP-2 (72 kDa) was found to be expressed in 11 samples as

**Table 1** Levels of type-IV collagenases (measured as square pixels per microgram of protein) in rectal cancer specimens

| | **Preradiotherapy MMP expression (kDa)** | | | | **Post-radiotherapy MMP expression (kDa)** | | | |
|---|---|---|---|---|---|---|---|---|
| **Patient No** | **92** | **82** | **72** | **62** | **92** | **82** | **72** | **62** |
| 1 | 51.7 | ND | 38.2 | 47.7 | 219.9 | ND | 39.6 | 74.1 |
| 2 | 227.4 | ND | 55 | 52.3 | 61.7 | ND | 53 | 53 |
| 3 | 53 | ND | 21.1 | 12.6 | 91.5 | ND | 61.8 | 128 |
| 4 | 474.3 | ND | 304.6 | 371.3 | 1817.3 | ND | 396.5 | 780 |
| 5 | 390 | ND | 24 | 30 | 578 | ND | 38 | 48 |
| 6 | 390 | ND | ND | ND | 803 | ND | 93 | 414 |
| 7 | 6150 | ND | 458 | 408 | 3465 | ND | 290 | 466 |
| 8 | 2312 | ND | 135 | 188 | 5831 | ND | 384 | 127 |
| 9 | 711 | ND | 116 | ND | 1635 | ND | 578 | 163 |
| 10 | 757 | ND | 116 | 96 | 4232 | ND | 816 | 750 |
| 11 | 146 | ND | ND | ND | 407 | ND | ND | ND |
| 12 | 278 | ND | ND | ND | 1145 | ND | 31 | 90 |

ND, not detected.

compared to nine samples, preradiotherapy, with a twofold increase in the levels ($P = 0.042$, Figure 2). Post-radiotherapy, 11/12 cancers expressed active MMP-2 (62 kDa) as compared to 8/12 cancers before the radiation treatment. There was three- to fourfold increase (Figure 2) in the levels of the enzyme ($P < 0.01$).

### Normal mucosa

All seven normal rectal mucosal samples expressed pro MMP-9 enzyme while pro MMP-2 was expressed in 6/7 samples before radiotherapy. There was virtually no expression of any active form either before or after the radiotherapy. Figure 1B shows a zymogram with no active forms of either of type IV collagenases detected.

In contrast to the changes seen in the rectal cancer, radiotherapy did not affect the expression of pro MMP-9 in normal mucosa while a non significant reduction in the levels of pro MMP-2 was observed ($P = 0.70$).

Immunocytochemistry performed on the tissue sections (Figure 3 A–D) localized the enzymes to the stromal cells rather than the tumour cells.

### DISCUSSION

Surgery remains the primary treatment for rectal cancer and, while this has become more refined, there is still some debate over the need for adjuvant treatment. The aim of preoperative high-dose radiotherapy is to sterilize the resection margins and to destroy microscopic collections of cancer cells situated outside the mesorectum, in presacral nodes and at lateral pelvic walls, a potential focus of local recurrence, and a source of distant metastasis. A significant number of patients still suffer from local recurrence (11% in the Swedish Rectal Cancer Trial, 1997) and distant metastasis even after adjuvant radiotherapy.

There is some evidence that irradiation leads to the induction of type-IV collagenases in vitro (Sheela et al, 1986; Sawaya et al, 1994). These observations prompted us to investigate whether similar changes take place in the in vivo situation. Our results demonstrate that preoperative high-dose radiotherapy leads to a significant increase in levels of MMP-2 and MMP-9 in rectal cancer tissue, with

 *© 2000 Cancer Research Campaign*

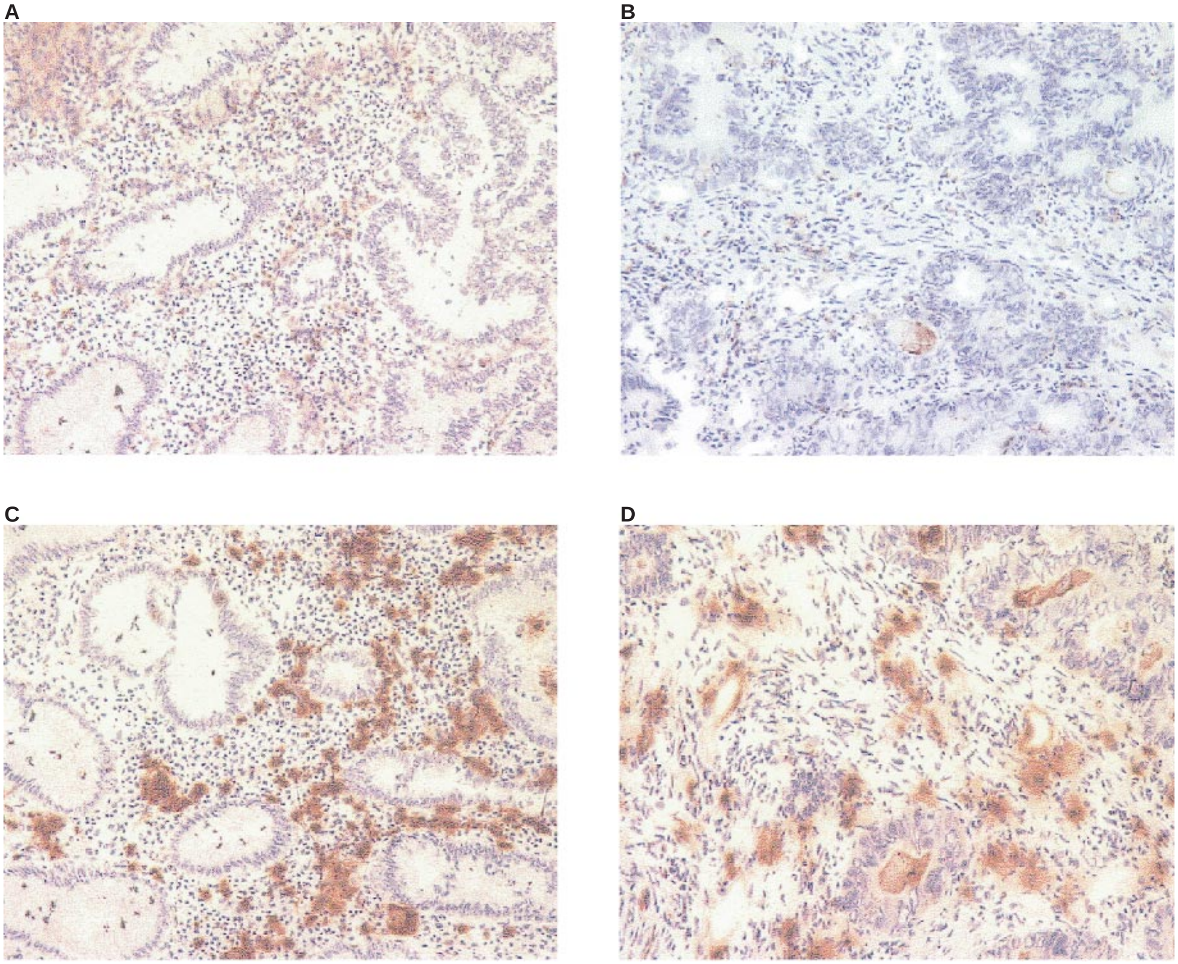

**Figure 3** Immunocytochemistry with anti-MMP-2 antibody. (**A**) Tumour preradiotherapy and (**B**) tumour post-radiotherapy (magnification × 100). Immunocytochemistry with anti-MMP-9 antibody. (**C**) Tumour preradiotherapy and (**D**) tumour post-radiotherapy (magnification × 100)

no increase observed in normal mucosa. A significant three- to fourfold increase of activated MMP-2 was also seen after radiotherapy. We have previously demonstrated via gelatin zymography (Parsons et al, 1998) that it is the active form of MMP-2, which is associated with the malignant phenotype of colorectal neoplasms. In another study (Azzam et al, 1993), poor prognosis in breast cancer correlated not with the expression of pro MMP-2 (72 kDa) but with its activated form (62 kDa). Localization studies using immunocytochemistry or in situ hybridization (Poulsom et al, 1992; Pyke et al, 1993; Zeng et al, 1995; Tomita et al, 1996) have shown that MMP-2 and MMP-9 are localized to the stromal cells at the growing edge of the tumour rather than in tumour cells themselves. It has been suggested (Pyke et al, 1993) that recruitment of stromal cells to assist cancer cells in the invasive process may be mediated through signals, in the form of various cytokines, from the cancer cells. The increased levels of MMP-2 and MMP-9 in rectal tumour tissue seen after radiotherapy may be due to stromal activation by cytokines produced by the cancer cells in response to the treatment. This remains to be determined by future studies which should include immunocytochemistry to localize type-IV collagenase expression in conjunction with cytokines such as TGF-$\alpha$ and -$\beta$, IL-1, TNF-$\alpha$ and EGF. Activation of MMP-2 occurs at the surface of the cancer cell by membrane type MMP (MT1-MMP) (Strongin et al, 1995; Kinoshita et al, 1996). The increased activity of activated MMP-2 seen after adjuvant radiotherapy may be due to overexpression or activation of MT-1-MMP.

There is considerable evidence to indicate that colorectal cancers overexpress type-IV collagenases (Zeng et al, 1995; Liabakk et al, 1996; Parsons et al, 1996; Tomita et al, 1996) and utilize these enzymes to establish a favourable environment for growth and invasion. The proteolytic activity of the enzymes helps the tumour to establish an invasive edge, promoting entry into and out of blood or lymphatic vessels and expansion of a metastatic tumour at the secondary site (Chambers and Matrisian, 1997). Reports have provided strong evidence of a crucial role for MMPs in the process of tumour angiogenesis (Johnson et al, 1994; Vu et al, 1998), suggesting another route in which MMPs influence the growth and behaviour of tumour cells. Mice that lack MMP-9, show failure of vascularization and apoptosis in the skeletal growth plate early in development (Vu et al, 1998). When these mice are crossed with animals in which expression of MMP-9 coincides with activation of an angiogenic switch during tumour

*© 2000 Cancer Research Campaign*

formation, loss of MMP-9 suppresses tumorigenesis. This animal data has revealed evidence linking MMP-9 causally with angiogenesis. In the present study radiotherapy led to a significant two- to threefold increase in levels of MMP-9. The MMP inhibitors TIMP-1 and TIMP-2, which specifically inhibit MMP-9 and MMP-2 respectively, have been shown to be anti-angiogenic, strongly suggesting that these two MMPs play an important role in angiogenesis (Albini et al, 1994).

Our report provides the first evidence that preoperative high-dose radiotherapy leads to increased type-IV collagenase activity in rectal cancer. This may be responsible for re-establishing a blood supply and resurrecting the invasive edge of remaining viable cancer cells in the pelvis, enhancing the recurrence rate. Our findings suggest that inhibition of type-IV collagenases may be a useful therapeutic adjunct to radiotherapy in patients with resectable rectal cancer, although prospective trials are needed to determine the actual clinical benefits.

### ACKNOWLEDGEMENTS

The authors wish to thank Derby City General Hospital for funding this study and Mr JR Reynolds and Mr RI Hall, Consultant Surgeons at Derby City General Hospital, for allowing us to include their patients in the study.

### REFERENCES

Abulafi AM and Williams SN (1994) Local recurrence of rectal cancer: the problem, mechanism, management and adjuvant therapy. *Br J Surg* **81:** 7–19

Albini A, Fontanini G, Masiello L, Tacchetti C, Bigini D, Luzzi P, Noonan DM and Stetler-Stevenson WG (1994) Angiogenic potential in vivo by Kaposi's sarcoma cell-free supernatants and HIV-1 *tat* product: inhibition of KS-like lesions by tissue inhibitor of metalloproteinase-2. *AIDS* **8**: 1237–1244

Azzam H, Arand G, Lippman M and Thompson E (1993) Association of MMP-2 activation potential with metastatic progression in human breast cancer cell lines independent of MMP-2 production *J Natl Cancer Inst* **85**: 1758–1764

Adam IJ, Mohamdee MO, Martin IG, Scott N, Finan PJ, Johnston D, Dixon MF and Quirke P (1994) Role of circumferential margin in the local recurrence of rectal cancer. *Lancet* **344**: 707–711

Boag AH and Young ID (1994) Increased expression of 72-kDa type-IV collegenase in prostate adenocarcinoma. *Am J Path* **144**: 585–591

Ballin M, Gomez DE, Sinha CC and Thorgeirsson UP (1988) Ras oncogene mediated induction of a 92 kDa metalloproteinase; strong correlation with the malignant phenotype. *Biochem Biophys Res Commun* **154**: 832–838

Brown PD, Bloxidge RE, Anderson E and Howell A (1993) Expression of activated gelatinase in human invasive breast cancer. *Clin Exp Metastasis* **11**: 183–189

Chambers AF and Matrisian LM (1997) Changing views of the role of matrix metalloproteinases in metastasis. *J Natl Cancer Inst* **89**: 1260–1270

Cedermark B, Johansson H, Rutqvist LE, Wilking N and Stockholm Colorectal Cancer Group (1995) The Stockholm 1 trial of pre-operative short-term radiotherapy in operable rectal carcinoma. A prospective randomised trial. *Cancer* **75**: 2269–2275

Chandler S, Coates R, Gearing A, Lury J, Wells G and Bone E (1995) Matrix metalloproteinases degrade myelin basic protein. *Neurosci Lett* **201**: 223–226

Davies B, Waxman J, Wasan H, Abel P, Williams G, Krausz T, Neal D, Thomas D, Hanby A and Balkwill F (1993*a*) Levels of matrix metalloproteinases in bladder cancer correlate with tumour grade and invasion. *Cancer Res* **53**: 5365–5369

Davies B, Miles D, Happerfield L, Naylor M, Bobrow L, Rubens R and Balkwill F (1993*b*) Activity of type-IV collagenase in benign and malignant breast disease. *Br J Cancer* **67**: 1126–1131

Duffy MJ and McCarthy K (1998) Matrix metalloproteinases in cancer: prognostic markers and targets for therapy (Review). *Int J Oncol* **12**: 1343–1348

Heussen C and Dowdle (1980) Electrophoretic analysis of plasminogen activators in polyacrylamide gels containing sodium dodecyl sulphate and copolymerised substrates. *Anal Biochem* **102**: 196–202

Johnson MD, Kim HC, Chesler L, Tsao-Wu G, Bouck N and Polverini PJ (1994) Inhibition of angiogenesis by tissue inhibitor of metalloproteinase. *J Cell Phys* **160**: 194–202

Jaziorka M, Haboubi NY, Schofield PF, Ogata Y, Nagase H and Woolley DE (1994) Distribution of gelatinase B (MMP-9) and type IV collagen in colorectal carcinoma. *Int J Colorectal Dis* **9**: 141–148

Kinoshita T, Sato H, Takino T, Itoh M, Akizawa T and Seiki M (1996) Processing of a precursor of 72-kDa type IV collegenase/gelatinase A by a recombinant membrane type 1 matrix metalloproteinase. *Cancer Res* **56**: 2535

Kleiner D and Stetler-Stevenson W (1994) Quantitative zymography: detection of picogram quantities of gelatinases. *Anal Biochem* **218**: 325–329

Liabakk N, Talbot I, Smith RA, Wilkinson K and Balkwill F (1996) Matrix metalloproteinase 2 (MMP-2) and matrix metalloproteinase 9 (MMP-9) type IV collegenases in colorectal cancer. *Cancer Res* **56**: 190–196

Moll UM, Youngleib GL, Rosinski KB and Quigley JP (1990) Tumour promoter-stimulated $M_r$ 92 000 gelatinase secreted by normal and malignant human cells: Isolation and characterisation of the enzyme from HT1080 tumour cells. *Cancer Res* **50**: 6162–6170

Moriya Y, Hojo K, Sawada T and Koyama Y (1989) Significance of lateral node dissection for advanced rectal carcinoma at or below the peitoneal reflection. *Dis Colon Rectum* **32**: 307–315

Marsh PJ, James RD and Schofield PF (1994) Adjuvant pre-operative radiotherapy for locally advanced rectal carcinoma. *Dis Colon Rectum* **37**: 1205–1214

Murphy G and Docherty AJP (1992) The matrix metalloproteinases and their inhibitors. *Am J Resp Cell Mol Biol* **7**: 120–125

Muller D, Wolf C, Abecassis J, Millon R, Engelmann A, Bronner G, Rouyer N, Rio MC, Eber M, Methlin G, Chambon P and Basset P (1993) Increased stromelysin 3 gene expression is associated with increased local invasiveness in head and neck squamous cell carcinoma. *Cancer Res* **53**: 165–169

Meyers MH and Ries LA (1989) Cancer patient survival rates: SEER program results for 10 years of follow-up. *CA Cancer J Clin* **39**: 21–32

Nakajima M, Morikawa K, Fabra A Bucana CD and Fidler IJ (1990) Influence of organ environment on extracellular matrix degradative activity and metastasis of human colon carcinoma cells. *J Natl Cancer Inst* **82**: 1890–1898

Poulsom R, Pignatelli M, Stetler-Stevenson WG, Liotta LA, Wright PA, Jeffery RE, Longcroft JM, Rogers L and Stamp GWH (1992) Stromal expression of 72 kDa type-IV collegenase (MMP-2) and TIMP-2 mRNA in colorectal neoplasia. *Am J Pathol* **141**: 389–396

Pyke C, Ralfkiaer E, Tryggvason K and Dano K (1993) Messenger RNA for two type IV collegenases is located in stromal cells in human colon cancer. *Am J Pathol* **142**: 359–365

Parsons SL, Watson SA, Collins HM, Griffin NR, Clarke PA and Steele RJC (1998) Gelatinase (MMP-2 and -9) expression in gastrointestinal malignancy. *Br J Cancer* **78**: 1495–1502

Quirke P, Durdey P, Dixon MF and Williams NS (1986) Local recurrence of rectal adenocarcinoma due to inadequate surgical resection. Histopathological study of lateral tumour spread and surgical excision. *Lancet* **ii**: 996–999

Seir CFM, Kubben FJGM, Ganesh S, Heerding MM, Griffioen G, Hanemaaijer R, vanKrieken JHJM, Lamers CBHW and Verspaget HW (1996) Tissue levels of matrix metalloproteinases MMP-2 and MMP-9 are related to overall survival of patients with gastric carcinoma. *Br J Cancer* **74**: 413–417

Sawaya R, Tofilon PJ, Mohanam S, Ali-Osman F and Liotta LA (1994) Induction of tissue-type plasminogen activator and 72-kDa type-1V collegenase by ionizing radiation in rat astrocyte. *Int J Cancer* **56**: 214–218

Sheela S and Kennedy RA (1986) Radiation-induced anchorage-independent growth and collegenase production in diploid human fibroblasts. *Carcinogenesis* **7**: 201–205

Strongin AY, Collier I, Bannikov G, Marmer BL, Grants GA and Goldberg GI (1995) Mechanism of cell surface activation of 72-kDa type IV collegenase. *J Biol Chem* **270**: 5331–5338

Swedish Rectal Cancer Trial (1997) Improved survival with pre-operative radiotherapy in resectable rectal cancer. *N Engl J Med* **336**: 980–987

Stetler-Stevenson WG, Liotta LA and Kliener DE (1993) Extracellular matrix 6: role of matrix metalloproteinases in tumour invasion and metastasis. *FASEB J* **7**: 1434–1441

Turpeenniemi-Hujanen T, Thorgeirsson UP, Hart IR, Grant SS and Liotta LA (1985) Expression of collegenase IV (basement membrane collegenase) activity in murine tumour cell hybrids that differ in metastatic potential. *J Natl Cancer Inst* **75**: 99–103

Tomita T and Iwata K (1996) Matrix metalloproteinases and tissue inhibitors of metalloproteinases in colonic adenomas–adenocarcinomas. *Dis Colon Rectum* **39**: 1255–1264

Takahashi K, Mulliken JB, Kozakewich HP, Rogers RA, Folkman J and Ezekowitz RA (1994) Cellular markers that distinguish the phases of hemangioma during infancy and childhood. *J Clin Invest* **93**: 2357–2364

© 2000 Cancer Research Campaign

Urbanski SJ, Edwards DR, Hershfield N, Huchcroft SA, Shaffer E, Sutherland L and Kossakowska AE (1993) Expression pattern of metalloproteinases and their inhibitors changes with the progression of human sporadic colorectal neoplasia. *Diag Mol Pathol* **2**: 81–89

Vu TH, Shipley JM, Bergers JE, Helms JA, Hanahan D, Shapiro SD, Senior RM and Werb Z (1998) MMP-9/Gelatinase B is a key regulator of growth plate angiogenesis and apoptosis of hypertrophic chondrocytes. *Cell* **93**: 411–422

Yamagata S, Ito Y, Tanaka R and Shimizu S (1988) Gelatinases of metastatic cell lines of murine colonic carcinoma as detected by substrate-gel electrophoresis. *Biochem Biophys Res Commun* **151**: 158–162

Yamagata S, Yoshii Y, Suh JG, Tanaka R and Shimizu S (1991) Occurrence of an active form of gelatinase in human gastric and colorectal carcinoma tissues. *Cancer Lett* **59**: 51–55

Zucker S, Lysik RM, Zarrabi MH and Moll U (1993) $M_r$ 92 000 type IV collagenase is increased in plasma of patients with colon cancer and breast cancer. *Cancer Res* **53**: 140–146

Zeng ZS, Huang Y, Cohen AM and Guillem G (1996) Prediction of colorectal cancer relapse and survival via tissue levels of matrix metalloproteinase-9. *J Clin Oncol* **14**: 3133–3140

Zeng ZS and Guillem JG (1995) Distinct pattern of matrix metalloproteinase 9 and tissue inhibitor of metalloproteinase I mRNA expression in human colorectal cancer and liver metastasis. *Br J Cancer* **72**: 575–582

© 2000 Cancer Research Campaign
